# Placing Objects on Table Is Preferred over Direct Handovers When Users Are Occupied

**DOI:** 10.3390/s25072140

**Published:** 2025-03-28

**Authors:** Thieu Long Phan, Akansel Cosgun

**Affiliations:** School of IT, Deakin University, Melbourne, VIC 3125, Australia; paul.phan@deakin.edu.au

**Keywords:** human–robotinteraction, object handovers, service robots

## Abstract

Service robots commonly deliver objects through direct handovers, assuming users are fully attentive. However, in real-world scenarios, users are often occupied with other tasks. This paper investigates how user attentiveness affects preferences between direct handovers and placing objects on a table. A user study was conducted (n = 25) to evaluate these strategies in scenarios where participants were either occupied (simulated via a typing task) or unoccupied. Results show that placing objects on the table significantly enhances user experience when users were occupied, with higher ratings for satisfaction, perceived safety, confidence in robot ability and intuitiveness of interaction. While direct handovers performed better with unoccupied users compared to occupied users, table placement maintained consistently high performance regardless of user state. All participants preferred table placement when occupied, and the majority preferred it even when unoccupied. These findings suggest table placement should be the default object delivery strategy for service robots, particularly in environments where user attention may vary. We also discuss implications for robot design and propose future directions for adaptive delivery behaviors.

## 1. Introduction

The ability to deliver objects is a critical skill for service robots operating in human environments. While mobile robots without manipulators can perform basic delivery tasks, they require users to actively retrieve objects from them (Unhelkar et al. [1], Chung and Cakmak [2]). Robots equipped with arms, on the other hand, can directly hand over objects to users, with robot-to-human handovers being the predominant delivery method (Ortenzi et al. [3]). However, much of the existing research assumes that users are fully attentive and expecting the delivery, overlooking real-world scenarios where users may be occupied with other tasks.

Previous studies have explored how user context influences object delivery preferences. Through a video survey, Martinson et al. [4] found that user activity impacts preferred handover methods: users favored approaching the robot while walking, direct handovers while standing or talking, and place-to-table delivery while working or eating. Building on this, Quispe et al. [5] proposed learning individual user preferences for handover methods. Other research has demonstrated specific indirect delivery scenarios, such as Odabasi et al. [6]’s system for placing refilled water bottles on tables.

Surprisingly, only two studies have directly compared direct and indirect handovers through user experiments, published 15 years apart. Both focused on individuals with specific needs: Choi et al. [7] worked with motor-impaired users, while Langer et al. [8] worked with visually impaired users. The assumption in the literature that direct handovers are generally most suitable for object delivery may explain the lack of comparative studies on indirect handovers involving the general population. These studies revealed interesting patterns: user preferences were evenly divided in Choi’s work, while in Langer’s study, the majority of participants preferred indirect handovers, citing that table-placed objects were easier to locate. Notably, both studies reported higher success rates for indirect handovers.

Our work addresses a critical gap in the literature by examining how user attentiveness influences delivery method preferences in everyday scenarios. Specifically, we investigate preferences between direct handovers and place-to-table delivery in an office setting where users may be engaged in work tasks. Drawing on Martinson et al. [4]’s findings, we hypothesize that users will prefer place-to-table delivery when occupied. To test this, we conducted a user study comparing direct handover and place-to-table delivery across two conditions: occupied (simulated via a typing game) and unoccupied (sitting idle). Participants evaluated their experience across multiple dimensions including satisfaction, safety, intuitiveness, confidence in the robot and mental demand using Likert scales. To our knowledge, this is the first systematic comparison of direct and indirect handovers with occupied users in a controlled study, offering novel insights into designing more effective and user-centered robotic delivery systems. This paper elaborates the results presented in our preliminary work (Phan and Cosgun [9]).

## 2. Implementation

Our system implementation focuses on developing reliable navigation and delivery capabilities for comparing handover methods in a controlled environment. Below, we detail the key components and their implementation.

### 2.1. Robot Platform

We utilized the Hello Robot Stretch 2 platform, a mobile manipulator designed for human environments. The robot features a manipulator with prismatic joints offering a 52 cm range of motion and a 2-degrees-of-freedom end effector. The robot is equipped with a 340-degree field of view 2D laser scanner, used for localization and navigation.

### 2.2. Waypoint Navigation

**Figure 1:**
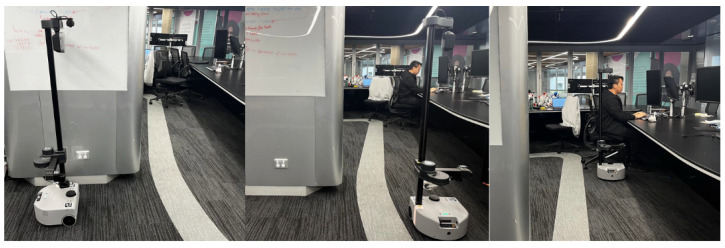
Robot progression through experiment waypoints. **Left:** Starting position, hidden from participant’s view. **Middle:** Intermediate waypoint. **Right:** Goal waypoint where handover is performed.

To enable autonomous navigation, we implemented a simple waypoint navigation approach using the Robot Operating System (ROS2). While our approach supports handling a sequence of waypoints, only two waypoints were defined for this experiment, as shown in Figure 1. The robot starts from a hidden position out of the participants’ view, which ensures that participants are not initially aware of the robot (left image). It then moves to an intermediate waypoint (middle image) before reaching the final waypoint (right image) for object delivery. At the final waypoint, the robot performs an in-place rotation towards the delivery point, ensuring accurate positioning for either handover or placing the object on the table.

The robot moves in a straight line between waypoints, executing an in-place rotation at each waypoint to reorient itself toward the next waypoint. This predictable motion ensured consistency and avoided the complexities of the ROS2 navigation stack, which often follows curved paths and exhibits unpredictable behaviors, such as stopping or performing in-place rotations as recovery actions. While we implemented obstacle detection that would halt the robot if triggered, this safety feature was not activated during any experimental trials. The simplified navigation strategy helped participants focus on the handover interaction rather than the robot’s approach.

### 2.3. Delivery Methods

Handover: In this method, the robot positions itself near the participant’s right side and raises its arm to 85 cm—approximately shoulder height for seated individuals. This height was chosen to optimize ergonomic reach without requiring significant posture adjustments. The robot extends its arm toward the participant and maintains this position until receiving a manual signal from the research team upon successful object transfer. The robot then retracts the arm, rotates back and returns to its starting position.

Placing the object on the table: The robot follows the same initial positioning and arm height procedures as the handover method but places the object on the table in front of the participant. This approach allows participants to retrieve the object at their convenience. After placement, the robot retracts its arm and returns to its starting position.

## 3. User Study Design

### 3.1. Setup and Preparation

We recruited 25 participants aged 18 to 24 years from the university student population. Participants viewed a 4-min tutorial video (https://www.youtube.com/watch?v=gXDYLUsjjOs, accessed on 6 December 2024) explaining the experiment’s objectives, tasks, and delivery methods. The video detailed trial procedures, feedback requirements, and demonstrated both delivery methods. Participants then practiced the typing game used to simulate the occupied condition, with instructions to maximize their typing scores to ensure high task engagement. The study received approval from the Deakin University Ethics Board with identifier SEBE-2024-29.

### 3.2. Independent Variables

The study employed a 2 × 2 within-subjects design with two independent variables:

1. Delivery method:
  - Handover: Direct object transfer to participant.
  - Table placement: Object placed on table for retrieval.
2. User State:
  - Unoccupied: Full attention available for robot interaction.
  - Occupied: Engaged in typing game during delivery.

**Figure 2:**
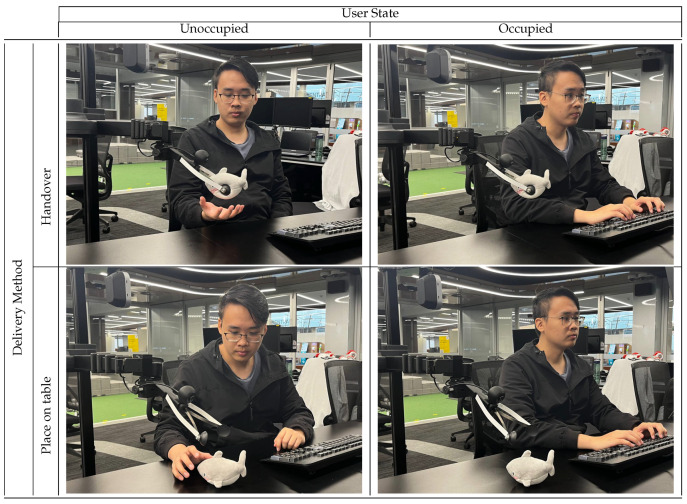
Overview of handover trial conditions categorized by method and user attentiveness.

Each participant experienced all four condition combinations (Figure 2). We counterbalanced condition order across participants to minimize ordering effects. During pilot studies, it was observed that participants were uncertain about the delivery method after the robot started moving. This uncertainty occasionally caused inefficiencies. For example, participants expecting the object to be placed on the table might delay their actions while waiting for the placement to occur. Conversely, participants expecting a normal handover might prematurely extend their hand for the object. To address this, we placed visible labels indicating the current method (direct/indirect) near participants.

### 3.3. Study Hypotheses

We formulated five hypotheses about the interaction between delivery methods and user state:

**H1:**  *User experience for direct handover will be better in the unoccupied versus occupied state, as unoccupied users can better engage in the synchronous interaction required for handovers.*

**H2:**  *User experience for table placement will not be worse in the occupied state compared to unoccupied state, since this method allows users to retrieve objects at their convenience without disrupting their current task.*

**H3:**  *For occupied users, table placement will provide better user experience than direct handover, as it minimizes interruption of their primary task and allows flexible object retrieval timing.*

**H4:**  *For unoccupied users, direct handover will provide better experience than table placement, as it offers immediate object transfer when users are fully available to interact.*

**H5:**  *Table placement will have a larger positive effect on user experience for occupied versus unoccupied users, reflecting its particular value in minimizing task disruption for busy users.*

### 3.4. Questionnaires

To evaluate participants’ experiences during the study, a structured questionnaire was designed. The questionnaire aimed to capture various dimensions of the human–robot interaction. These included participants’ prior experiences with robots, their subjective evaluations of handover quality, and their preferences for delivery methods. Questions were categorized into three stages: pre-experiment, per-trial feedback and post-trial preferences as in Table 1, Table 2 and Table 3. Feedback for the per-trial survey, the primary survey in this user study, was collected using a 7-point Likert scale.

These questions captured participants’ immediate impressions of the handover process, allowing for a detailed analysis of satisfaction, safety, intuitiveness, confidence, and cognitive load.

These questions aimed to identify participants’ ultimate preferences for delivery methods depending on their level of engagement.

## 4. Results

This section presents our analysis of two delivery methods under occupied and unoccupied conditions, addressing the five hypotheses from Section 3.3.

Statistical analysis revealed significant effects of delivery method and user attentiveness on several key dimensions of user experience, as summarized in Table 4. A statistical significance threshold of 0.05 was used for all *p*-values. Satisfaction, safety, intuitiveness, and confidence in the robot’s ability all varied significantly across conditions, while confidence in the robot’s ability showed moderate variation. Mental demand, however, did not show statistically significant differences across conditions, suggesting that neither delivery method nor user attentiveness imposed additional cognitive load. Consequently, we excluded mental demand from further analysis.

### 4.1. Direct Handovers (H1)

Mean survey scores for handovers, comparing occupied and unoccupied conditions, are presented in Table 5. Results indicate that for handovers, the unoccupied condition resulted in higher satisfaction, perceived safety, and intuitiveness. Confidence, although slightly lower in significance than other metrics, did reach statistical significance (*p* = 0.039). All four metrics now statistically support the hypothesis, affirming H1.

### 4.2. Table Placement (H2)

Mean survey scores for placing the object on the table, comparing occupied and unoccupied conditions, are presented in Table 6. Results indicate that user experience for having the object placed on the table was not significantly different between occupied and unoccupied conditions across all metrics. Specifically, satisfaction, safety, intuitiveness and confidence scores all showed *p*-values well above the threshold, confirming that user experience in occupied scenarios was comparable to unoccupied ones.

The results support **H2**, which hypothesized that user experience would not be worse in occupied conditions compared to unoccupied conditions.

### 4.3. Occupied Users (H3)

Mean survey scores for handovers and object placements on the table under unoccupied conditions are presented in Table 7. Results indicate that participants reported significantly higher satisfaction, safety and intuitiveness for placing the object on the table compared to having it handed over directly in occupied conditions, with all three metrics showing statistically significant differences. Confidence, while also higher for placing the object on the table than for handovers, reached statistical significance (*p* = 0.008).

The post-trial question “When you are occupied, which type of handover do you prefer?” further supports these findings, with all participants indicating a preference for having the object placed on the table under occupied conditions. This alignment between subjective preferences and survey results reinforces the observed trend.

With all four metrics showing statistically significant differences, the results strongly confirm **H3**, supporting the hypothesis that user experience is better for placing the object on the table compared to handovers during occupied conditions.

### 4.4. Unoccupied Users (H4)

Mean survey scores for handing the object over directly and placing the object on the table in the unoccupied condition are presented in Table 8. Results indicate that participants reported slightly higher satisfaction, safety and confidence when having the object placed on the table compared to having it handed over directly. Among these, only the safety metric showed a statistically significant difference, while confidence approached significance and satisfaction showed a modest trend. Intuitiveness scores, in particular, were nearly identical for both conditions, indicating no discernible advantage for either handover method according to this metric.

The typing game results, as reflected in participants’ scores, further support these findings, showing that placing the object on the table allowed participants to maintain focus on their primary task, whereas handovers were more disruptive. This aligns with the post-trial question “When you are unoccupied, which type of handover do you prefer?” where 77% of participants favored having it placed on the table. This discrepancy between subjective preferences and the mixed statistical results suggest that other factors, such as perceived efficiency or task expectations, may influence user preferences under unoccupied conditions.

Overall, the results do not support **H4**, which hypothesized that user experience would be better for handovers compared to placing the object on the table when participants were unoccupied. Instead, the findings indicate a potential advantage for placing the object on the table, particularly in terms of safety and reduced task disruption, aligning with participant preferences.

### 4.5. Comparative Benefit of Table Placement (H5)

Hypothesis H5 posited that placing the object on the table would have a larger positive effect on user experience metrics when participants were occupied compared to when they were unoccupied. To test this hypothesis, we analyzed mean scores for satisfaction, safety, intuitiveness and confidence for placing the object on the table across occupied and unoccupied conditions. The results, presented in Table 9, reveal no statistically significant differences were observed between the two conditions, although all metrics continued to show consistently high performance across both states.

Although no significant differences were observed, the descriptive statistics highlight consistent high performance for placing the object on the table across both conditions. For instance, satisfaction scores in the occupied condition were similar to those in the unoccupied condition. A similar pattern was observed for safety and confidence. Interestingly, intuitiveness scores were slightly higher in the occupied condition than in the unoccupied condition, although this difference also did not reach statistical significance.

These findings suggest that having the object placed on the table is robust across both attentiveness states, maintaining consistently high user experience scores regardless of user busyness. While the hypothesis anticipated a stronger positive effect in the occupied condition, the lack of significant differences indicates that placing the object on the table performed equally well in both contexts. This consistency may reflect the method’s ability to reduce cognitive and physical demands on participants while minimizing disruptions.

Overall, the results partially support **H5**. Although the expected amplified benefit of having the object placed on the table in the occupied condition was not observed, the method’s consistent performance across varying levels of user attentiveness demonstrates its robustness and suitability for diverse scenarios.

## 5. Discussion

The user study results suggest that hypotheses **H1**, **H2**, **H3**, and **H5** were affirmed. **H4** was not supported, with results favoring placement of the object on the table over handovers in unoccupied conditions.

### 5.1. Implications for Handover Strategies

The results emphasize the importance of the placement of objects on the table to enhance user experience in occupied scenarios. By reducing cognitive interruptions, having the object placed on the table allows users to focus on their primary tasks, significantly enhancing subjective metrics and improving objective metrics (typing game score). These findings align with existing research emphasizing the importance of minimizing task disruptions during human–robot interactions in real-world contexts Mainprice et al. [10], Fehér et al. [11]. Additionally, placing objects on the table supports adaptive human–robot interaction principles, catering to varying user attentiveness levels while maintaining seamless integration into daily activities Fehér et al. [11], Maeda et al. [12], Néron and Teulière [13].

In unoccupied scenarios, both handover and placing the object on the table demonstrated comparable effectiveness in terms of satisfaction and intuitiveness. However, the marginal safety advantage of placing the object on the table reinforces its potential as a default strategy in dynamic environments, where user availability and situational demands may fluctuate unpredictably Néron and Teulière [13], Nikolakis et al. [14]. These results align with insights from Fehér et al. [11], Maeda et al. [12] on socially aware navigation systems, which emphasize safety as a priority alongside task efficiency.

The results underscore the need for robots to adopt flexible handover strategies that dynamically adapt to user attentiveness. In high-priority scenarios, such as delivering critical items, handing the object over directly may remain essential. However, placing objects on the table provides a consistent and non-intrusive alternative for less time-sensitive interactions. Studies like those by Maeda et al. Maeda et al. [12] and Mainprice et al. Mainprice et al. [10] similarly advocate for dynamic robot behavior to balance user needs with task demands.

From a robot design perspective, these insights can guide the development of handover systems optimized for varying user states. For robots capable of both direct object handover and placement of the object on the table, an ideal strategy involves adapting the delivery method based on the user’s attentiveness: place objects on the table during occupied periods to minimize disruption, and perform handover during unoccupied periods to ensure efficiency. Conversely, robots limited to handing the object over directly should consider deferring the task until users are unoccupied, reducing the cognitive and task performance impacts observed during in occupied state. This approach aligns with Lee et al. Lee et al. [15], who emphasized the importance of timing in delivering visual cues or proactively attracting user attention. Visual attention is a critical factor in human–robot interaction (HRI) and has been shown to improve task success during collaborative activities Admoni et al. [16], Moon et al. [17]. However, excessive anthropomorphic design can backfire, as Onnasch et al. Onnasch and Hildebrandt [18] found that overly human-like robots may distract users from tasks.

Lessons learned from this study may also apply to human-to-robot handovers. For instance, Strabala et al. Strabala et al. [19] suggested that robots could indicate readiness to receive objects by purposefully adjusting their gaze. However, work on indirect human-to-robot handovers remains limited, with Choi et al. Choi et al. [20] being a notable exception in predicting object placement locations during indirect interactions.

### 5.2. Insights from Post-Study Interviews

Post-study interviews provided valuable qualitative insights into participant experiences. Many participants highlighted the consistency and reliability of having the object placed on the table, noting that this approach allowed them to focus on their primary task without the need to actively engage with the robot. Placement of the object on the table was perceived as less intrusive, making it particularly suitable for scenarios involving multitasking or cognitive load. As one participant remarked, “I liked how I could just continue working without worrying about when the object was delivered”.

Conversely, the handovers were appreciated for their immediacy and convenience when users were not preoccupied. However, several participants expressed concerns about the level of precision required to interact with the robot during handovers. For example, some participants mentioned feeling anxious about grasping the object incorrectly or inadvertently interrupting the robot’s operation. This concern highlights the potential stress that handovers may impose, especially in scenarios where the user feels rushed or uncertain.

A few participants also suggested that the robot’s behavior when handing the object over directly could be improved by incorporating more explicit cues, such as verbal or visual signals, to guide their actions during object retrieval. Others noted that while placing the object on the table was generally preferable for occupied scenarios, it might lack the immediacy required for high-priority items, such as medicine or a ringing phone. These reflections underscore the importance of tailoring handover strategies to the specific context and user needs.

## 6. Conclusions

This study examined the effectiveness of direct handovers and table placement when users are occupied or unoccupied, offering insights into optimizing human–robot interaction strategies. Results demonstrate that table placement significantly enhanced user satisfaction, safety and intuitiveness in occupied conditions by reducing disruptions and allowing users to focus on primary tasks. In unoccupied scenarios, both methods were effective, with table placement showing a slight safety advantage, reinforcing its versatility as a reliable default strategy.

Despite its contributions, the study has limitations. The small participant pool limits the generalizability of the findings. Additionally, the occupied condition, simulated using a typing game, may not fully capture the complexities of real-world multitasking in dynamic environments. The use of a neutral object also excludes potential variations in user preferences for critical items, such as medications or urgent deliveries, where direct handovers might be preferred. These findings underscore the importance of adaptive, context-aware handover strategies to improve service robot usability and acceptance.

We strongly believe that human–robot object handovers should be field-tested in real-world situations. Our work, by considering user attentiveness, is a first step towards this direction.

Future research can explore user state detection to dynamically assess user attentiveness and adapt handover strategies in real time. Expanding studies to include diverse user demographics, task complexities and dynamic settings (e.g., homes, offices, warehouses) is essential for understanding the scalability and robustness of adaptive handover mechanisms. Additionally, contexts with multiple users or simultaneous tasks introduce unique challenges that could further refine these strategies.

## Figures and Tables

**Table 1:** Pre-experiment survey question.

Question
Have you previously interacted with robots? Yes/No/Maybe)

**Table 2:** Per-trial survey questions.

Question
How satisfied were you with the handover? (Very Dissatisfied-Very Satisfied)
How safe did you feel during the handover with the robot? (Very Unsafe–Very Safe)
How intuitive was your interaction with the robot? (Very Confusing–Very Intuitive)
How confident are you in the robot’s ability to safely and accurately handle objects? (Not Confident at All–Very Confident)
How mentally demanding was the interaction with the robot? (Not at All Demanding–Extremely Demanding)

**Table 3:** Post-trial survey questions.

Question
When you are occupied, which type of handover do you prefer? (Direct Handover/Indirect Handover)
When you are unoccupied, which type of handover do you prefer? (Direct Handover/Indirect Handover)

**Table 4:** ANOVA results showing the effects of delivery method and user attentiveness across study metrics. Statistically significant results are indicated by *.

Metric	F-Value	*p*-Value
Satisfaction level	21.45	$1.8\times 10^{-9}$ *
Safety level	18.32	$3.2\times 10^{-8}$ *
Intuitiveness level	14.70	$5.5\times 10^{-7}$ *
Confidence in robot’s ability	4.25	0.010 *
Mental demand	1.03	0.410

**Table 5:** Handovers: Mean survey scores comparing occupied versus unoccupied. Statistically significant results are indicated by *.

Metric	Occupied	Unoccupied	*p*-Value
Satisfaction	4.49	6.38	$4.2\times 10^{-4}$ *
Safety	4.52	6.33	$7.1\times 10^{-4}$ *
Intuitiveness	4.43	6.19	$9.8\times 10^{-4}$ *
Confidence	5.55	6.18	0.039

**Table 6:** Place on table: Mean survey scores comparing occupied versus unoccupied. None of the results were statistically significant.

Metric	Occupied	Unoccupied	*p*-Value
Satisfaction	6.65	6.70	0.503
Safety	6.68	6.74	0.472
Intuitiveness	6.45	6.30	0.121
Confidence	6.42	6.48	0.636

**Table 7:** Occupied condition: Mean survey scores comparing handover and placement on table. Statistically significant results are indicated by *.

Metric	Handover	Place on Table	*p*-Value
Satisfaction	4.49	6.65	$3.8\times 10^{-5}$ *
Safety	4.52	6.68	$2.5\times 10^{-5}$ *
Intuitiveness	4.43	6.45	$3.0\times 10^{-5}$ *
Confidence	5.55	6.42	0.008 *

**Table 8:** Unoccupied condition: Mean survey scores comparing handover and placement on table. Statistically significant results are indicated by *.

Metric	Handover	Place on Table	*p*-Value
Satisfaction	6.38	6.70	0.085
Safety	6.33	6.74	0.012 *
Intuitiveness	6.19	6.30	0.720
Confidence	6.18	6.48	0.059

**Table 9:** Comparative benefit of placing the object on the table: Mean survey scores comparing occupied and unoccupied conditions.

Metric	Occupied	Unoccupied	*p*-Value
Satisfaction	6.65	6.70	0.503
Safety	6.68	6.74	0.472
Intuitiveness	6.45	6.30	0.121
Confidence	6.42	6.48	0.636

## Data Availability

The per-trial survey results are anonymized and made available online at https://drive.google.com/file/d/1aw5F1wmuQy9CyowPKVCBWQZWBbkdLAI5/view?usp=sharing (accessed on 11 January 2025).
